# Novel approach to modeling high-frequency activity data to assess therapeutic effects of analgesics in chronic pain conditions

**DOI:** 10.1038/s41598-021-87304-w

**Published:** 2021-04-08

**Authors:** Zekun Xu, Eric Laber, Ana-Maria Staicu, B. Duncan X. Lascelles

**Affiliations:** 1 Department of Statistics, North Carolina State University, Raleigh, NC USA; 2 Comparative Pain Research and Education Center, College of Veterinary Medicine, North Carolina State University, Raleigh, NC USA; 3 Translational Research in Pain (TRiP) Program, North Carolina State University, College of Veterinary Medicine, Raleigh, NC USA; 4 Thurston Arthritis Center, UNC School of Medicine, Chapel Hill, NC USA; 5 Center for Translational Pain Research, Department of Anesthesiology, Duke University, Durham, NC USA

**Keywords:** Neuroscience, Computational biology and bioinformatics, Computational models, Data processing, Statistical methods

## Abstract

Osteoarthritis (OA) is a chronic condition often associated with pain, affecting approximately fourteen percent of the population, and increasing in prevalence. A globally aging population have made treating OA-associated pain as well as maintaining mobility and activity a public health priority. OA affects all mammals, and the use of spontaneous animal models is one promising approach for improving translational pain research and the development of effective treatment strategies. Accelerometers are a common tool for collecting high-frequency activity data on animals to study the effects of treatment on pain related activity patterns. There has recently been increasing interest in their use to understand treatment effects in human pain conditions. However, activity patterns vary widely across subjects; furthermore, the effects of treatment may manifest in higher or lower activity counts or in subtler ways like changes in the frequency of certain types of activities. We use a zero inflated Poisson hidden semi-Markov model to characterize activity patterns and subsequently derive estimators of the treatment effect in terms of changes in activity levels or frequency of activity type. We demonstrate the application of our model, and its advance over traditional analysis methods, using data from a naturally occurring feline OA-associated pain model.

## Introduction

To anyone who suffers chronic and persistent musculoskeletal pain, the negative impact on their quality of life is constant. In the U.S., more than 100 million people (nearly one third of the population) suffer from persistent pain with an associated economic cost of $600 billion USD annually; this cost exceeds that of cardiovascular disease, cancer, and diabetes combined^1^. The most significant contribution to this cost comes from the impact of osteoarthritis (OA) and other musculoskeletal pain^1^. OA results in the deterioration of all components of joints^2^ and is often associated with pain. OA-associated pain results in significant morbidity and economic costs^3^; these costs, an aging population, and the growing knowledge of the health and psychological benefits of maintaining mobility and activity, have made the treatment of OA and the associated pain a public health priority^4–8^. However, recent review papers show that the current practice of translational biomedical research is not producing new therapeutics for pain control in humans^9–11^. While these reviews highlight the lack of translation of basic research into new approved therapeutics for treatment of persistent pain in humans, they also discuss how the processes involved could be optimized to improve the chances of successful translation, including discussion of improved models and more relevant outcome measures. As OA and associated pain affects all mammals, the study of OA in non-human animals is both important in its own right (to increase the function and quality of life in affected animals) and for its ability to generate new knowledge about the treatment of OA in humans. Recently, the use of naturally occurring OA in pet animals has been suggested as a means of helping to improve translational research for the development of analgesic therapeutics in humans^12^. A significant advantage of naturally occurring disease in pets as a model of human conditions is the variation and complexity of the model. Measurement of spontaneous activity as an outcome measure may be particularly relevant to translational work as spontaneous activity may relate to spontaneous pain—something that has been difficult to model in animals. Just as the variability in naturally occurring disease is an advantage, the variability in clinically meaningful outcome measures such as activity is a challenge. This could be the reason for the lack of use of activity as a functional outcome measure in pain studies in humans, despite the importance of mobility and activity to quality of life^7^. The work we are reporting is motivated by our involvement in studies of OA-associated pain and the treatment effects on mobility and activity in domestic cats^13,14^. We posit that the methodology we describe presents a significant advance in how such activity data are analyzed when used as an outcome measure and has relevance to both large animal models and clinical evaluation in humans.

The current study uses data from a randomized cross-over study designed to evaluate the effectiveness of meloxicam, a nonsteroidal anti-inflammatory drug (NSAID), in owned, pet adult domestic cats with OA-associated pain^15^. In this study, cat activity patterns were measured at one-minute intervals using an omnidirectional accelerometer^16^. Accelerometer readings are integers quantifying the intensity of change in acceleration over the preceding, pre-defined epoch. Thus, for each subject, the accelerometer produces high-frequency, integer-valued, longitudinal data. The main goal of the present study is to re-evaluate the use of and analysis of activity data to determine whether meloxicam is effective in reducing OA-associated pain. We aimed to use the objectively measured physical activity recorded via accelerometers and analyze these data using a novel zero-inflated hidden Poisson semi-Markov model. The primary hypothesis of the present study is that meloxicam reduces OA-associated pain and this manifests through increased activity during more intense activity during the treatment period relative to the placebo period. One common approach for the analyses of such data is to aggregate the observed accelerometer data within each subject and treatment condition and subsequently to use an ANOVA to compare treatment with control^15–17^. Alternatively, one could aggregate the data over a shorter time interval, e.g., a day, and model the aggregated process using methods for smooth longitudinal data^18^. However, such aggregation can obscure changes in behavior patterns which do not produce a large difference in mean activity. For example, an effective treatment that reduces pain may lead to higher quality rest (more zero activity readings), but also more high-intensity movement (more high activity readings), potentially producing no change in mean activity. High volume activity data has the potential to be a more sensitive outcome measure, but thus far, analysis of such complex, high-volume multidirectional effects on activity, that takes into account the wide variation in individual activity patterns, has not been developed.

We propose modeling the minute-by-minute accelerometer data using a hidden semi-Markov model. Such a model is scientifically appealing in that the hidden states can be viewed as corresponding to latent (unobserved) activities, e.g., running, walking, resting, etc., and state duration corresponds to the length of time a subject is engaged in a given activity. Latent state-space models are common in the analysis of mobility data measured using wearable computing^19–23^. In the context of treatment evaluation, hidden Markov models have also been used to model latent health states and subsequently conduct inference for activity patterns in terms of transitions among these states and activity patterns within each state^24,25^; however, previous applications have considered relatively coarse time scales, e.g., daily or weekly measurements, and subsequently low data volume. In the current application, subject activity is measured every minute and as a result each subject has more than one-hundred thousand measurements during the observation period. Such high volume allows for flexible modeling and estimation of distinct parameters for each subject; this is important as activity patterns can vary widely across subjects.

In the current example of the spontaneous cat OA-pain model, more than 70% of the observations are zero and therefore we propose a zero-inflated hidden Poisson semi-Markov model with patient-specific intensities in each latent state. The proposed model is an extension of a zero-inflated Poisson hidden Markov model^26,27^ to the hidden semi-Markov model framework that allows for distinct state duration densities^28–30^. Furthermore, to facilitate computationally efficient estimation with high-frequency data, we propose a two stage estimation procedure^31,32^ that can be used with millions of observations without specialized computing resources.

The data for this work come from a study of the treatment effects of meloxicam (a non-steroidal anti-inflammatory drug) in a feline model of spontaneous OA-associated pain^15,33^. This study was a randomized, double-blind, placebo-controlled, crossover study to evaluate the effectiveness of meloxicam treatment to improve mobility and function in owned, pet cats with OA-associated pain. This study was approved by the Animal Care and Use Committee (Protocol # 11-102-O) at North Carolina State University College of Veterinary Medicine (NCSU-CVM), and written owner consent was granted in each case following verbal discussion of the study.

## Results

**Figure 1:**
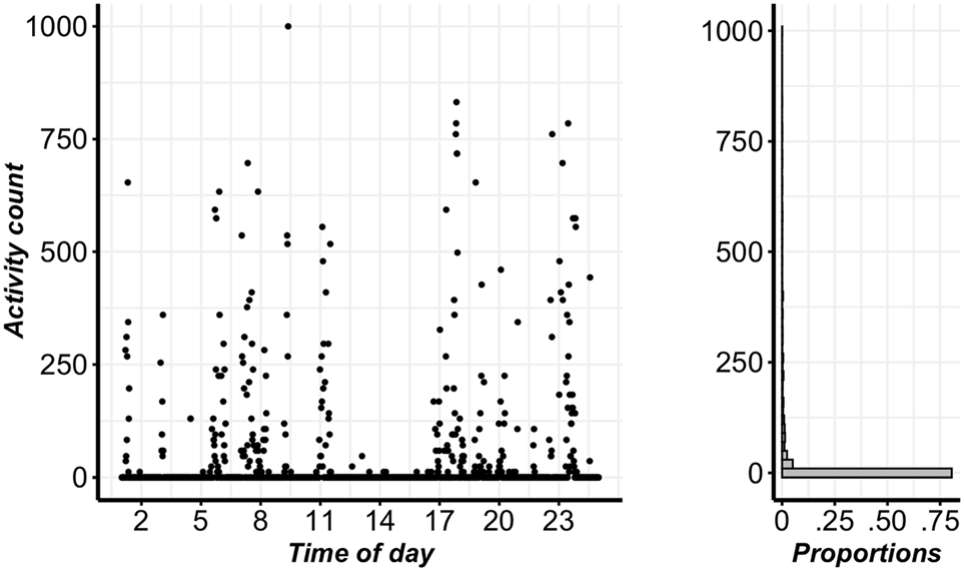
Left: Sample daily accelerometer data for a typical cat in the study of the treatment effect of meloxicam, showing the crepuscular pattern of activity expected in cats. Right: Histogram showing the proportions of time spent in different levels of activity during a 24 h period.

Figure 1 displays the daily activity profile for a typical subject. There are multiple prolonged intervals in which there is no movement. In total, more than 70% of the minute-by-minute activity counts are zero; furthermore, the non-zero counts are heavily right-skewed owing to infrequent but high-intensity activities. Thus, such cat accelerometer data are heavy-tailed with an excessive proportion of zeros.

**Figure 2:**
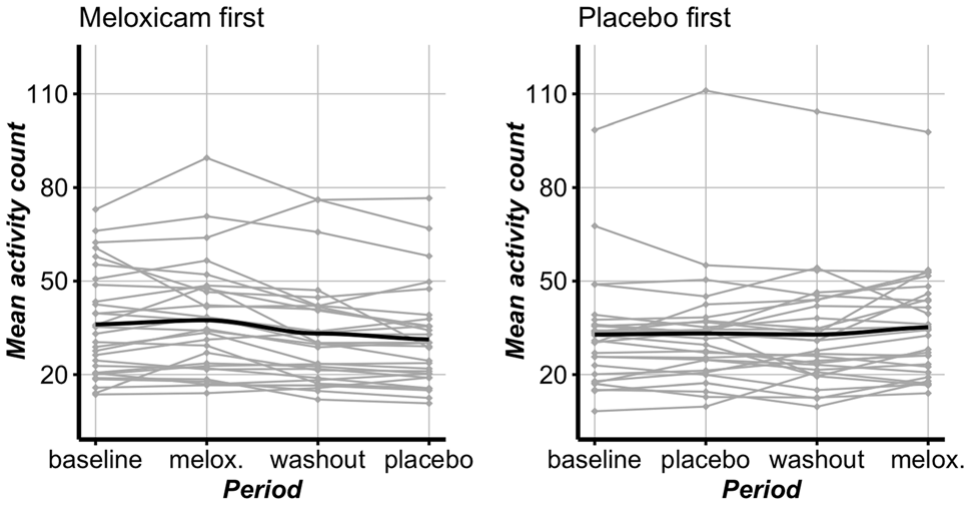
Plots of the mean activity count over each period for each subject separated by treatment group. The dark line in each panel is the group mean activity count.

Figure 2 displays mean activity counts for each subject across each period of the study. In this figure, each gray line shows the mean activity count for a single subject in the study, and the black line shows the overall mean activity count for all subjects in their associated treatment group. The left panel shows data for the subjects that were randomized to meloxicam in the first treatment period, whereas the right panel shows data for the subjects that were randomized to placebo in the first treatment period. In both panels, one can see that the overall mean activity count is higher under meloxicam than placebo, but only marginally. A total of 41 out of 58 cats have a numerically larger mean activity count in the meloxicam period than in the placebo period. However, there is large between-subject variability. The standard errors for the group mean activity count at each treatment period range from 16.7 to 18.1.

We applied the proposed zero-inflated hidden semi-Markov model (detailed in the Methods section) to the longitudinal accelerometer data from this study of the treatment effect of meloxicam. We chose the number of latent activity states to be six using minimum Bayesian information criterion (BIC) as our data-driven selection method. The covariates included in the model were: treatment status, weekend (yes/no), night (yes/no), sex, Body Condition Score (BCS), age, total OA score, and treatment sequence.

**Figure 3:**
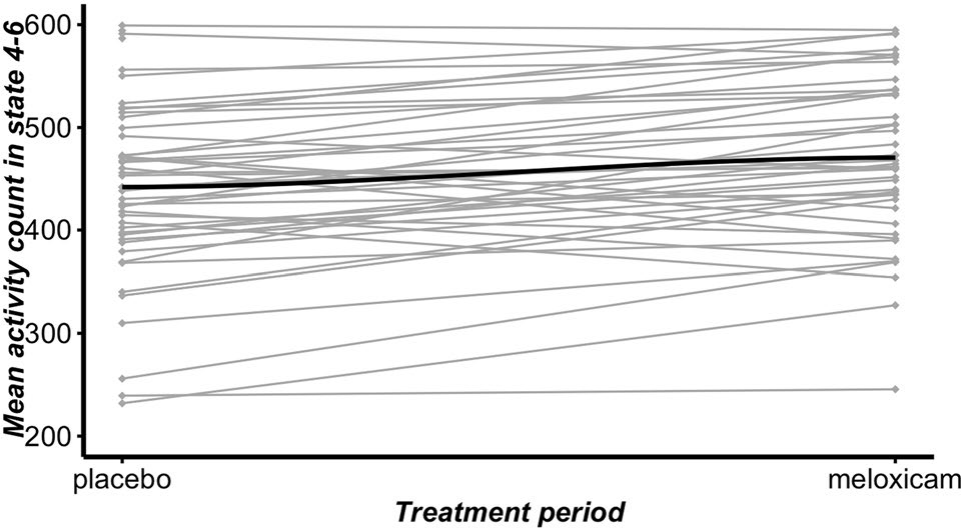
Mean activity count for each subject in the placebo and meloxicam treatment period for latent state 4–6. The dark line in each panel is the overall mean activity count. The mean increase in the activity count is 23.0, with a standard error of 7.2.

Table 1 displays the estimated treatment effects in terms of mean activity counts within each latent state. There are significant effects (*p* < 0.05) in latent states 4–6 suggesting that cats treated with meloxicam may have increased mean activity while engaged in more intensive activities (e.g., running, jumping, etc.). The effect size of the treatment on those more intensive activities ranges from 7.28 to 9.14%. Table 2 shows the mean proportion of time spent in each latent state estimated using the Viterbi algorithm^34^. It can be seen that the proportion of time spent in each activity is similar across the treatment and placebo periods. The proportion of time spent is each latent activity states is similar before and after the meloxicam treatment (Table 2); what significantly changes is the mean activity counts in more intense states (4–6), i.e., cats become more active in those intense activity states (Table 1). The mean activity count for each subject in the placebo and meloxicam treatment period for latent state 4–6 is shown in Fig. 3.

**Table 1:** Summary of treatment effects (meloxicam vs. placebo) in each latent activity state in the increasing order of mean activity count.

Parameter	Estimate	Standard error	*p* value
State 1: odds of zero	− 0.0299	0.0433	0.4899
State 1: mean activity count	0.0222	0.0181	0.2187
State 2: mean activity count	0.0444	0.0311	0.1538
State 3: mean activity count	0.0524	0.0318	0.0990
State 4: mean activity count	0.0914	0.0351	0.0091
State 5: mean activity count	0.0819	0.0235	0.0005
State 6: mean activity count	0.0728	0.0260	0.0051

Actual activities represented in each state are unknown, but for instance, state 1 may include resting and other activities with the lowest mean activity count, while state 6 may include jumping and other activities with the highest mean activity count. At significance level 0.05, there is significant positive treatment effect in state 4, 5, 6, i.e. higher level activity intensity states.

**Table 2:** Mean proportion of time spent in each decoded activity state. The latent states are in increasing order of mean activity count.

Activity state	Meloxicam (%)	Placebo (%)
State 1	83.6	84.5
State 2	5.5	5.3
State 3	4.5	4.3
State 4	3.4	3.3
State 5	2.1	1.9
State 6	0.9	0.8

Actual activities represented in each state are unknown, but for instance, state 1 may include resting and other activities with the lowest mean activity count, while state 6 may include jumping and other activities with the highest mean activity count. At significance level 0.05, there is significant positive treatment effect in state 4, 5, 6, i.e. higher level activity intensity states.

**Figure 4:**
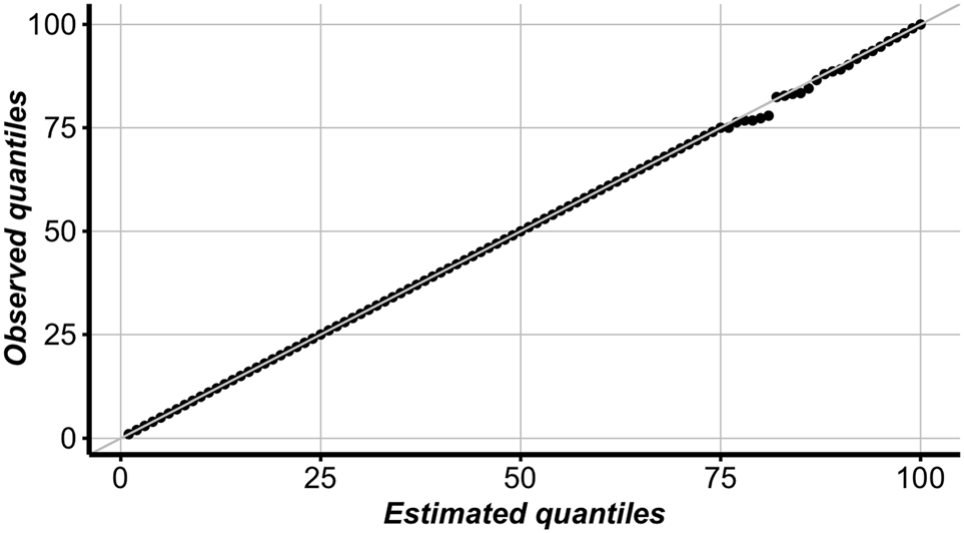
Estimated quantiles based on fitted model versus observed quantiles. In both distributions, from the 1st up to the 75th quantiles are zero. The rest of the quantiles also match well with each other, which indicates there is no lack of fit in the proposed model.

Figure 4 shows a QQ-plot of the estimated quantiles using the fitted model and the observed quantiles of the activity counts. It can be seen that they follow each other closely and that there are no indications of serious lack-of-fit.

## Discussion

We proposed a zero-inflated Poisson hidden semi-Markov model for activity data captured by accelerometer readings in a naturally occurring model of OA-associated pain. The proposed model can be used to characterize the impact of treatment on activity including changes in activity duration, intensity, and frequency. In the context of OA-associated pain, understanding such impacts is critical to fully understanding the effects of treatment on patient mobility and therefore quality of life.

Our method presents a significant advance in the analysis of therapeutic efficacy using activity counts as compared to the original analysis in Gruen et al^15^ as well as subsequent work in Gruen et al^18^. In Gruen et al^15^, the activity counts were averaged within each treatment period for each cat, and an ANOVA model was used to determine the effectiveness of meloxicam. Their analysis showed a non-significant increase (*p* = 0.31) in the activity counts among the cats that received meloxicam in the first treatment period. This may be due to the effect of data aggregation method, which can smooth away important effects and reduce statistical power. Gruen et al^18^ aggregated the counts across days and then used functional data analysis methods to formally assess treatment effect. This method failed to identify a significant difference between normal and arthritic cats (*p* = 0.541) due to the same limitations discussed above.

In contrast, our proposed subject-specific method not only preserves the original data ‘as is’ but also accounts for the large between-subject variability. More importantly, our approach can analyze the conditional treatment effect in each activity state. Indeed, our model found that meloxicam significantly improves the mobility of cats in more intensive activities, while the activity patterns did not change in less intensive activities. Although an easy assumption is that the increase in more intense activities is beneficial, the clinical relevance of these findings is, at present, unknown.

Our approach is relevant to humans. Cats, like people, show patterns of activity, with large periods of relative or absolute inactivity. Cats are generally crepuscular—that is, they show phasic patterns of activity, as do humans. Such zero-inflated patterned activity is thus common to cats and humans, and so our approach is relevant to human activity data. As the approach does not need to know what activity is being performed, it negates the need for algorithms to detect specific activities.

Future work could support our approach by evaluating concurrent video capture of moving subjects to verify that as more intense activities are being performed, the relevant latent states change. Additionally, research is needed to define the clinical relevance of the changes we have detected using the zero-inflated hidden Poisson semi-Markov model.

Our refinement of the analysis of high-volume activity data in this naturally occurring model of pain provides a clinically relevant outcome measure that can be used in naturally occurring models osteoarthritis in companion animals to provide highly relevant data on the potential efficacy of putative drugs prior to Phase II and III efficacy studies in humans, thus improving the translational paradigm by optimizing the critical go/no-go decision prior to Phase II. Such sophisticated approaches to high-volume activity data have not been applied to humans and our approach also has relevance to the analysis of activity data in humans to better understand the impact of therapeutics in a more refined manner.

## Methods

The original study was approved by the Animal Care and Use Committee (Protocol # 11–102-O) at North Carolina State University College of Veterinary Medicine (NCSU-CVM), and written owner consent was granted in each case following verbal discussion of the study. The study was performed in accordance with the relevant guidelines and regulations. Full details of the study have been previously published^15^ and pertinent methodological information is provided in Supplementary File 1.

A total of 66 subjects were enrolled in the original study of which 58 had available accelerometer measurements from both arms of the cross-over design. Subjects were given placebo during the open label baseline period (Weeks 1 and 2). In the first blinded treatment period (Weeks 3, 4, and 5) half of the subjects were randomized to receive meloxicam and the remainder were randomized to receive placebo. At the end of the first blinded phase, subjects entered a three-week blinded washout (Weeks 6, 7, and 8) before switching treatments for the second blinded phase. All subjects wore an accelerometer (Actical) on their collar throughout the entire study, with the epoch set at 1 min, providing an activity count for every minute of the 11-week study for each subject.

### Zero-inflated Poisson hidden semi-Markov model

We assume that the observed data are of the form {(W_i_, Z_i_^1^, X_i_^1^, Y_i_^1^, …, Z_i_^T^, X_i_^T^, Y_i_^T^)} and comprise n independent replicates of the trajectory (W, Z^1^, X^1^, Y^1^, …, Z^T^, X^T^, Y^T^), where: $W \in {\mathbb{R}}^{d}$ denotes the baseline (pre-treatment) subject characteristics; T is a fixed time horizon; $Z^{t} \in {\mathbb{R}}^{p}$ denotes environmental factors at t = 1, …, T; $X^{t} \in {\mathbb{R}}^{q}$ encodes treatment administered at time t = 1, … ,T; and $Y^{t} \in {\mathbb{N}}$ denotes the integer accelerometer activity reading at time t = 1, … ,T. In our application, $Z^{t} \in \{ 0, 1\}^{2}$ comprises indicators coding period of day and weekend versus weekday, and $X^{t} \in \{ 0, 1\}^{1}$ encodes treatment indicators (active versus placebo). While we develop our models allowing for rather general environmental factors and treatment processes, having binary factors in our application makes it simpler to enforce a stochastic ordering on the distributions indexed by latent states which is important for interpretability and coherence of the fitted models, e.g., if one wants the latent states ordered by mean activity intensity within each subject and to have these latent states align across subjects. We model the evolution of the accelerometer data using a zero-inflated Poisson hidden semi-Markov model which develop in the remainder of this subsection.

Let $S_{i}^{t} \in \left\{ {1, \ldots , M} \right\}$ denote the latent state, i.e., the unobserved activity type, of subject i = 1, …, n at time t = 1, … ,T. At every single minute, each subject is assumed to be in one of the M latent activity states, with the convention that “1” represents a state where a low level of physical activity is typical; “2” represents a state where higher level activity is typical and so forth. The duration in each state is characterized by state-specific parameters and estimated from the data.

We assume the latent states evolve according to a semi-Markov model indexed by: (i) an initial state distribution, $\delta_{m,i} = P\left( {S_{i}^{1} = m} \right)$ for m = 1, …, M; (ii) state duration distributions $r_{m,i} \left( {v;x^{t} ,z^{t} } \right) = P(S_{i}^{t + 1} = m, \ldots ,S_{i}^{t + v} \ne m|S_{i}^{t} = m,X_{i}^{t} = x^{t} ,Z_{i}^{t} = z^{t} )$ for v > 0, m = 1, … , M; and (iii) the transition probabilities $Q_{i} \left( {m,l;x^{t} ,z^{t} } \right) = P{(}S_{i}^{t + 1} = l {|} S_{i}^{t} = m, S_{i}^{t + 1} \ne m, X_{i}^{t} = x^{t} ,Z_{i}^{t} = z^{t} )$ for $m, l = 1, \ldots ,M$. We model the initial state distribution nonparametrically using sample frequencies of the estimated latent states. We assume a multinomial logistic regression model for the transition probabilities so that

$$Q_{i} \left( {m,l;x^{t} ,z^{t} } \right) = \frac{{\exp \left( {d_{m,l,i}^{\prime } x^{t} + {\varrho }_{m,l,i}^{\prime } z^{t} } \right)}}{{\mathop \sum \nolimits_{k = 1}^{M} \exp \left( {d_{m,k,i}^{\prime } x^{t} + {\varrho }_{m,k,i}^{\prime } z^{t} } \right)}},$$

where $d_{m,k,i}$ and ${\varrho }_{m,k,i}$ are unknown coefficient vectors for m, k = 1, …, M. We assume that the state duration distribution follows a latent accelerated failure time model so that.

$$r_{m,i} \left( {v;x^{t} ,z^{t} } \right) = \exp \left( {c_{m,i}^{{\prime }} x^{t} + \eta_{m,i}^{{\prime }} z^{t} } \right)\mathop \smallint \limits_{v}^{v + 1} f_{m,i} \left\{ {\exp \left( {c_{m,i}^{{\prime }} x^{t} + \eta_{m,i}^{{\prime }} z^{t} } \right)u} \right\}du,$$

where $f_{m,i}$ is a base-density corresponding to a null treatment condition (coded as $x^{t} \equiv 0$) and $c_{m,i} , \eta_{m,i}$ are unknown coefficient vectors for m = 1, … ,M. We further assume that $Y_{i}^{t} \bot \left( {Z_{i}^{1} ,X_{i}^{1} ,S_{i}^{1} , \ldots , Z_{i}^{t - 1} ,X_{i}^{t - 1} ,S_{i}^{t - 1} } \right) | \left( {Z_{i}^{t} , X_{i}^{t} , S_{i}^{t} } \right)$ and that the accelerometer activity readings are distributed as.

$$P\left( {Y_{i}^{t} = y {|}S_{i}^{t} = 1,Z_{i}^{t} = z,X_{i}^{t} = x} \right) = p_{i}^{t} \left( {z,x} \right)I_{y = 0} + \left\{ {1 - p_{i}^{t} \left( {z,x} \right)} \right\}\frac{{\left\{ {\lambda_{i}^{t} \left( {m,z,x} \right)} \right\}^{y} {\rm{exp}}\left\{ {\{ \lambda_{i}^{t} \left( {m,z,x} \right)} \right\}}}{y!},$$

when the subject is in the latent state 1, and

$$P\left( {Y_{i}^{t} = y {|}S_{i}^{t} = m, Z_{i}^{t} = z,X_{i}^{t} = x} \right) = \frac{{\left\{ {\lambda_{i}^{t} \left( {m,z,x} \right)} \right\}^{y} {\rm{exp}}\left\{ { \lambda_{i}^{t} \left( {m,z,x} \right)} \right\}}}{y!},$$

when the subject is in latent state m = 2, 3, …, M. Thus, the accelerometer readings are modeled as a zero-inflated Poisson with intensity function $\lambda_{i}^{t} :S \times {\mathbb{R}}^{q} \times {\mathbb{R}}^{p} \to {\mathbb{R}}_{ + }$ and weight functions $p_{i}^{t} :S \times {\mathbb{R}}^{q} \times {\mathbb{R}}^{p} \to \left[ {0, 1} \right]$ for i = 1, …, n, t = 1, …, T. Furthermore, for each subject i, time t, and latent state m, we assume that these functions are of the form

$$\log \left\{ {\frac{{p_{i}^{t} \left( {z,x} \right)}}{{1 - p_{i}^{t} \left( {z,x} \right)}}} \right\} = b_{0,0,i} + b_{1,0,i}^{{\prime }} x + \gamma_{0,i}^{{\prime }}z,$$

$$\log \left\{ {\lambda_{i}^{t} \left( {m,z,x} \right)} \right\} = b_{0,m,i} + b_{1,m,i}^{{\prime }} x + \gamma_{m,i}^{{\prime }} z,$$

where $b_{0,k,i} ,b_{1,k,i} ,\gamma_{k,i} ,$ k = 0, 1, …, M are unknown coefficient vectors. In our application, in which $x^{t}$ and $z^{t}$ are represented as binary vectors, we impose the constraints $b_{0,m + 1,i} > b_{0,m,i} , b_{1,m + 1,i} \ge b_{1,m,i} ,\gamma_{m + 1,i} \ge \gamma_{m,i}$ for m = 1, …, M—1. These constraints ensure that the intensity functions of the latent states are monotone increasing, i.e., $\lambda_{i}^{t} \left( {m + 1, z,x} \right) > \lambda_{i}^{t} \left( {m,z,x} \right)$ for all z, x, and m.

The preceding model describes individual level accelerometer data as a function of evolving covariate and treatment information. The idea of using the framework of hidden Markov model to analyze physical activity data can also be seen in the recent work of Huang et al.^35^ and van Kuppevelt et al.^36^. However, the cat accelerometer data we consider are much sparser than the human accelerometer data in their work, which motivates a zero-inflation component in our model. Moreover, we can include exogenous, time-varying variables like treatment period, night indicator, and weekend indicator in our model so as to explicitly address their effects on the activity patterns of cats. Our individual level model is a nontrivial extension of the zero-inflated Poisson hidden Markov model in the literature^26,27^, which not only allows for distinct state duration densities but also incorporates covariates and treatment in both transition probabilities and latent state durations.

To draw more general, i.e., population-level, inferences we model individual-level treatment effect parameters as functions of baseline subject information as follows. We assume that ${\rm E}(b_{1,m,i } |W_{i} = w) = \varOmega_{m,0} + \varOmega_{m,1} w$, where $\varOmega_{m,0} \in {\mathbb{R}}^{q}$ and $\varOmega_{m,1} \in {\mathbb{R}}^{q \times d}$ are matrices of unknown coefficients encoding treatment effect on the outcome for m = 0, 1, …, M. Similarly, we assume that ${\rm{{E}(}}c_{m,i } {|}W_{i} = w) = \varGamma_{m,0} + \varGamma_{m,1} w$ and ${{{\rm E}(}}d_{m,l,i } {|}W_{i} = w) = \varLambda_{m,l,0} + \varLambda_{m,l,1} w$, where $\varGamma_{m,0} , \varLambda_{m,l,0} \in {\mathbb{R}}^{q}$ and where $\varGamma_{m,1} , \varLambda_{m,l,1} \in {\mathbb{R}}^{q \times d}$ encode the effect of treatment on state duration and transition probabilities for $m, l = 1, \ldots , M$. The idea of aggregating individual-level models to conduct population-level inference has been applied in other contexts including longitudinal and growth curve analyses^31,37,38^.

### Estimation

We estimate the subject-specific parameters using maximum-likelihood estimation via the forward–backward recursive representation of the likelihood^39^; the number of latent states, M, is selected using BIC^38^. Subsequently, the population-level parameters are estimated by regressing the subject-specific maximum likelihood estimators on baseline covariates using least squares. This two-stage approach, which will be detailed shortly, is computationally efficient for high-frequency data like those collected in the meloxicam study. Let

$$\theta_{i} = \left\{ {\delta_{m} , d_{m,k,i} ,\varrho_{m,k,i} , c_{m,i} ,\eta_{m,i,} ,b_{0,0,i} ,b_{0,m,i} ,b_{1,0,i} ,b_{1,m,i} ,\gamma_{0,i} ,\gamma_{m,i} } \right\}_{m,k = 1, \ldots ,M}$$

be the collection of subject-specific parameters for i = 1, …, n. We construct estimators $\hat{\theta }_{i,T}$ of $\theta_{i}$ by maximizing the log-likelihood (see supplementary file 2 for details).

For m = 0, 1, …, M, let $\hat{b}_{{1,{\rm{m}},{\rm{i}},{\rm{T}}}}$ denote the maximum likelihood estimator of $b_{1,m,i}$. Subsequently, define $\hat{\varOmega }_{m,0,n}$, $\hat{\varOmega }_{m,1,n} = argmin_{{\varOmega_{m,0} ,\varOmega_{m,1} }} \mathop \sum \limits_{i = 1}^{n} ||\hat{b}_{1,m,i,T} - \varOmega_{m,0} - \varOmega_{m,1} W_{i} ||^{2}$ to be the two-stage estimator of $\varOmega_{m,0} , \varOmega_{m,1}$. The estimators $\hat{\varGamma }_{m,0,n} ,\hat{\varGamma }_{m,1,n} ,\hat{\varLambda }_{m,l,0,n} ,\hat{\varLambda }_{m,l,1,n}$ of $\varGamma_{m,0} ,\varGamma_{m,1} ,\varLambda_{m,l,0} ,\varLambda_{m,l,1}$ are defined analogously.

The statistical analysis in the manuscript is implemented using the software R version 2.0.6. We developed an R package “ziphsmm” to analyze such high-volume, zero-inflated accelerometer data, which is publicly available online (https://cran.r-project.org/web/packages/ziphsmm/index.html).

### Theoretical properties

Throughout we use a star superscript, e.g.,$\theta_{i}^{*}$ to denote the population analogue of the maximum likelihood estimator of a parameter indexing the proposed model; if the model is correctly specified, then under the conditions stated below, these parameters will correspond to the true parameters indexing the generative model. In order to characterize the limiting behavior of the proposed estimators, we make the following assumptions for all $i = 1, \ldots ,n$ and $m, l = 1, \ldots ,M.$

(A0) The dimension of the subject-specific parameters is j, where j is known; $\theta_{i}^{*}$ is an interior point of $\Theta$, which is a compact subset of ${\mathbb{R}}^{j}$.
(A1) The state duration distributions $r_{m,i} \left( . \right)$ has finite support.
(A2) There exists $0 < \sigma_{ - } \le \sigma_{ + } < 1$ such that ${\upsigma }_{ - } \le P_{{\theta_{i }^{*} }} (S_{i}^{t + 1} = l|S_{i}^{t} = m) \le \sigma_{ + }$, for ${\rm{t}} = 1,{ } \ldots ,{\rm{T}}$; and $\mathop \sum \limits_{{{\rm{m}} = 1}}^{{\rm{M}}} P_{{\theta_{i}^{*} }} \left( {Y_{i}^{t} = y{|}S_{i}^{t} = m} \right) > 0$ for all ${\rm{y}} \in {\rm{supp Y}}_{{\rm{i}}}^{{\rm{t}}}$ and ${\rm{t}} = 1,{ } \ldots ,{\rm{T}}$.
(A3) For each ${\uptheta } \in {\Theta }$ the transition kernel indexed by ${\uptheta }$ is Harris recurrent and aperiodic^40–42^.
(A4) The transition kernel is continuous in ${\uptheta }$ in an open neighborhood of ${\uptheta }_{{\rm{i}}}^{*}$.
(A5) The hidden semi-Markov model is identifiable up to label-switching^43,44^.

These assumptions are standard in latent-state models^45–49^. Assumption (A0) avoids non-regularity occurring at boundary points; the assumption of a fixed dimension could be relaxed, for example, to the assumption that one has a strongly consistent estimator of j. Assumption (A1) is common in semi-markov models and simplifies asymptotic arguments; if the finite support condition does not hold, one can use a nested sequence of finite approximations at the expense of more delicate asymptotic arguments^50,51^. Assumption (A4) is a regularity condition that avoids non-standard asymptotic behavior associated with non-smooth functionals^52,53^. Assumptions (A2), (A3), (A5) ensure that the model is well-defined; we show that (A5) holds for the zero-inflated Poisson semi-Markov model in the supplementary material. A proof of the following lemma and theorem is also provided in the supplementary material.

Lemma 1. Under (A0)–(A5), the MLE for the subject-specific hidden semi-Markov model in the first stage is strongly consistent, $\hat{\theta }_{i,T} \to \theta_{i}^{*}$ almost surely as ${\rm{T}} \to \infty$ for all ${\rm{i}} = 1, \ldots ,{\rm{n}}.$

Define ${\Omega }_{{\rm{m}}}^{*} = \left[ {{\Omega }_{{{\rm{m}},0}}^{*} , \varOmega_{m,1}^{*} } \right]$ and the corresponding estimator $\hat{\varOmega }_{{{\rm{m}},{\rm{n}}}} = \left[ {\hat{\varOmega }_{{{\rm{m}},0,{\rm{n}}}} { },\hat{\varOmega }_{m,1,n} } \right].{ }$ Let ${\Gamma }_{{\rm{m}}}^{*} ,\hat{\varGamma }_{m,n} , \varLambda_{m,l} , \hat{\varLambda }_{m,l,n}$ be defined analogously. Further, define the augmented design matrix  $\tilde{W} \in {\mathbb{R}}^{{n \times \left( {d + 1} \right)}}$ whose ith row is $\widetilde{{W_{i} }} = \left[ {1, W_{i}^{^{\prime}} } \right] \in {\mathbb{R}}^{d + 1}$. Then the second stage regression models can be equivalently written as ${\rm{vec}}\left( {\hat{b}_{1,m,T} } \right) = \left( {I \otimes \tilde{W}} \right){\rm{vec}}\left( {\varOmega_{m} } \right) + \nu_{m} ,$ ${\rm{vec}}\left( {\hat{c}_{1,m,T} } \right) = \left( {I \otimes \tilde{W}} \right){\rm{vec}}\left( {\varGamma_{m} } \right) + \eta_{m}$, ${\rm{vec}}\left( {\hat{d}_{m,l,T} } \right) = \left( {I \otimes \tilde{W}} \right){\rm{vec}}\left( {\varLambda_{m,l} } \right) + \xi_{m,l}$, where vec is the vectorization operator, and ${\upnu }_{{\rm{m}}} , \eta_{m} , \xi_{m,l}$ are independent mean zero residuals. Define $\tilde{B} = {\rm{I}} \otimes \tilde{W} \in {\mathbb{R}}^{{{\rm{qn}} \times {\rm{q}}\left( {{\rm{d}} + 1} \right)}} { }$ whose ith row is $\tilde{B}_{i}$.

Theorem 3.2. Under (A0)–(A5), and further assume that ${\rm{H}} = {\rm{n}}^{ - 1} \mathop {\lim }\limits_{{{\rm{n}} \to \infty }} \tilde{W}^{{\prime }} W$ is a positive definite matrix. Then provided ${\rm{T}},{\rm{n}} \to \infty$ with $\frac{{\rm{n}}}{{\rm{T}}} \to 0$, each of the following converges in distribution to a Gaussian distribution with mean zero and identity covariance:

$$\begin{aligned} & \left\{ {\left( {\frac{1}{n}\tilde{B}^{{\prime }} \tilde{B}} \right)^{ - 1} {(}\frac{1}{n}\tilde{B}^{{\prime }} {\rm{diag}}\left[ {\left\{ {{\rm{vec}}\left( {\hat{b}_{1,m,T} } \right)_{i} - \tilde{B}^{{\prime }}_{i} {\rm{vec}}\left( {\hat{\varOmega }_{{m,{\rm{n}}}} } \right)} \right\}^{2} } \right]\left( {\frac{1}{n}\tilde{B}^{{\prime }} \tilde{B}} \right)^{ - 1} } \right\}^{{ - \frac{1}{2}}} \\ & \quad \times \sqrt {\rm{n}} \left\{ {{\rm{vec}}\left( {\hat{\varOmega }_{{{\rm{m}},{\rm{n}}}} } \right) - {\rm{vec}}\left( {{\Omega }_{{\rm{m}}}^{*} } \right)} \right\}, \\ & \quad \left\{ {\left( {\frac{1}{n}\tilde{B}^{{\prime }} \tilde{B}} \right)^{ - 1} {(}\frac{1}{n}\tilde{B}^{{\prime }} {\rm{diag}}\left[ {\left\{ {{\rm{vec}}\left( {\hat{c}_{m,T} } \right)_{i} - \tilde{B}^{{\prime }}_{i} {\rm{vec}}\left( {\hat{\varGamma }_{{m,{\rm{n}}}} } \right)} \right\}^{2} } \right]\left( {\frac{1}{n}\tilde{B}^{{\prime }} \tilde{B}} \right)^{ - 1} } \right\}^{{ - \frac{1}{2}}} \\ & \quad \times \sqrt {\rm{n}} \left\{ {{\rm{vec}}\left( {\hat{\varGamma }_{{{\rm{m}},{\rm{n}}}} } \right) - {\rm{vec}}\left( {{\Gamma }_{{\rm{m}}}^{*} } \right)} \right\}, \\ & \quad \left\{ {\left( {\frac{1}{n}\tilde{B}^{{\prime }} \tilde{B}} \right)^{ - 1} {(}\frac{1}{n}\tilde{B}^{{\prime }} {\rm{diag}}\left[ {\left\{ {{\rm{vec}}\left( {d_{m,l,T} } \right)_{i} - \tilde{B}^{{\prime }}_{i} {\rm{vec}}\left( {\hat{\varLambda }_{{m,{\rm{l}},{\rm{n}}}} } \right)} \right\}^{2} } \right]\left( {\frac{1}{n}\tilde{B}^{{\prime }} \tilde{B}} \right)^{ - 1} } \right\}^{{ - \frac{1}{2}}} \\ & \quad \times \sqrt {\rm{n}} \left\{ {{\rm{vec}}\left( {\hat{\varLambda }_{{{\rm{m}},{\rm{l}},{\rm{n}}}} } \right) - {\rm{vec}}\left( {{\Lambda }_{{{\rm{m}},{\rm{l}}}}^{*} } \right)} \right\}, \\ \end{aligned}$$

for all $${\rm{m}},{\rm{l}} = 1, \ldots ,{\rm{M}}.$$

In the preceding theorem, the requirement that $\frac{{\rm{n}}}{{\rm{T}}} \to 0$ is natural in applications using high-frequency accelerometer data where the number of observations per subject can be several orders of magnitude larger than the number of subjects. In the supplementary file 2, we have included extensive simulation experiments to evaluate the performance of the proposed treatment effect estimator.

## Supplementary Information

Supplementary Information 1.

Supplementary Information 2.
