# Supplementary material for: Novel approach to modeling high-frequency activity data to assess therapeutic effects of analgesics in chronic pain conditions
Source: Sci Rep. 2021 Apr 8;11:7737. doi: 10.1038/s41598-021-87304-w (PMC8032701; doi:10.1038/s41598-021-87304-w)
Supplement: Supplementary file 1 — Supplementary Information 1. [file 41598_2021_87304_MOESM1_ESM.docx]

***Novel approach to modeling high-frequency activity data to assess therapeutic effects of analgesics in chronic pain conditions.***

*Supplementary File 1. Pertinent details*

*Animals*

Animals enrolled in the study were all client owned animals with naturally occurring chronic musculoskeletal disease.

*Inclusion and Exclusion Criteria*

Cats were eligible to participate in the study if they had a qualifying degree of owner-noted mobility/activity impairment, evidence of pain during manipulation of at least two joints or spinal segments during veterinary orthopedic evaluation, and radiographic evidence of degenerative joint disease in at least two of the painful joints or spinal segments. Cats were required to be greater than 1 year of age and weigh more than 1 kg. Other medical conditions were ruled out by careful review of the medical records, owner history, physical examination, complete blood count, serum biochemistry panels, and urinalysis. Cats with chronic kidney disease up to and including IRIS stage 2 were eligible. Cats of any body condition score (on a subjective scale of 1 to 9) {Laflamme, 1997 #12772} were eligible.

*Qualifying degree of owner-noted mobility/activity impairment*

A Client Specific Outcome Measures (CSOM) assessment was constructed to rate the degree of impairment. Owners rated their cat’s ability to perform each activity on a Likert type scale ranging from 4 (No Problem) to 0 (Impossible). Ratings were converted to numerical scores, with a possible range of 0-12 for the total score. In order to ensure that cats enrolled in the current study had moderate to severe activity impairments, as rated by their owners, cats were eligible for inclusion if they received an owner-rated score of ≤5 on Day 0. Owners completed the CSOM without knowledge of the cut-off for inclusion.

*Orthopedic and Radiographic evaluation*

Every joint and axial skeletal segment (cervical, thoracic, lumbar, and lumbo-sacral) was palpated and manipulated. A pain response score from 0 (no pain) -4 (severe) was assigned to each joint or axial segment based on the cat’s reaction, as previously described. {Gruen, 2017 #15153} A total pain score was calculated as the sum of all the individual appendicular joint and axial skeletal segment pain scores. Cats were sedated using a standard protocol and orthogonal radiographs were taken of every joint and spinal segment. Radiographs were reviewed for the presence of degenerative joint disease by a board-certified veterinary radiologist masked to the presence or location of pain. A radiographic severity score from 0 (normal) 10 (ankylosed) was assigned to each joint or axial segment, and summed scores represented the total OA score.

*Randomization method*

The cats were randomly allocated to one of two treatment sequences (meloxicam followed by placebo, OR, placebo followed by meloxicam), assigned according to predetermined randomization tables. Randomization was stratified by owner-rated degree of impairment based on total CSOM score.

*Sample Size Estimation*

Sample size estimation was based on previous FMPI results. Using an expected mean difference between groups of 0.3, and within patient standard deviation of 0.5, sample size estimation indicated that 61 patients would be needed for 90% power. Subjective owner assessments such as the FMPI have been found to be less sensitive than objective accelerometry and so a sample size estimation based on the FMPI was considered appropriate for evaluation of the data using accelerometry.

*Masking*

NCSU-CVM Pharmacy personnel packaged meloxicam or placebo for administration to each cat. Placebo was identical to drug, minus the active ingredient, meloxicam. Packaging for drug or placebo product was identical to prevent identification of treatment group by owners or investigators, and bottles were identified only by the days they were to be administered. Individual bottles for each period were delivered to the owner with both investigator and owner remaining masked to treatment group.

*Study timeline*

Cats were screened on Day 0. Eligible cats were enrolled and fitted with an activity monitor (AM; Actical Z, Philips Respironics) worn for the duration of the study. Owners were given placebo (unmasked) at 0.07 mL/kg/day to be given orally during the baseline period (Days 1-14). Following Day 14, all future treatments were masked and volume-matched. During treatment periods, cats received either placebo (0.07 ml/kg/day) or meloxicam (0.035 mg/kg/day). Following Day 14, there were 3 consecutive treatment periods: 1) placebo or meloxicam; 2) placebo; 3) meloxicam or placebo. As described above, all treatment periods were masked. Each treatment period was 3 weeks long (including treatment period 2, which was the blinded washout period).

*Drug*

The drug used (meloxicam) was commercially available (0.5mg/mL) supplied by the sponsor of the original study (Boeringher Ingelheim) (Carbopol^®^-based, benzoate-preserved, colloidal sol containing meloxicam at 0.05mg/mL). The placebo was supplied by Boeringher Ingelheim, and was identical to meloxicam, but without the active ingredient.

*Cases evaluated*

A total of 66 subjects were enrolled in the original study of which 58 had available accelerometer measurements from both arms of the cross-over design. Of the 66 subjects enrolled, 8 cats did not complete both arms of the study: 3 cats were withdrawn prior to the first treatment period due to owner non-compliance; 4 cats were withdrawn after the first treatment (2 for vomiting, 1 for behavior disorder, 1 for seizures); 1 cat was withdrawn at the end of the wash-out period due to acute kidney injury) (See figure 2 of {Gruen, 2015 #15175})
