# Supplementary material for: Novel approach to modeling high-frequency activity data to assess therapeutic effects of analgesics in chronic pain conditions
Source: Sci Rep. 2021 Apr 8;11:7737. doi: 10.1038/s41598-021-87304-w (PMC8032701; doi:10.1038/s41598-021-87304-w)
Supplement: Supplementary file 2 — Supplementary Information 2. [file 41598_2021_87304_MOESM2_ESM.pdf]

Supplementary material for:

Novel approach to modeling high-frequency activity data to assess therapeutic effects of analgesics in chronic pain conditions.

Zekun Xu, Eric B. Laber, Ana-Maria Staicu and B. Duncan X. Lascelles

This supplementary material contains three sections, which are: (1) the details of maximum likelihood estimation for zero-inflated Poisson hidden semi-Markov model; (2) the proof the theoretical results; (3) extensive simulations experiments to show that the proposed treatment effect estimator is consistent.

Maximum likelihood estimation for zero-inflated Poisson hidden semi-Markov model

We estimate the subject-specific parameters using maximum-likelihood estimation via the forward-backward recursive representation of the likelihood; the number of latent states, M, is selected using BIC. Subsequently, the population-level parameters are estimated by regressing the subject-specific maximum likelihood estimators on baseline covariates using least squares. This two-stage approach, which will be detailed shortly, is computationally efficient for high-frequency data like those collected in the meloxicam study. Let

$$\boldsymbol{\theta}_i = \{(\delta_m, \mathbf{d}_{m,k,i}, \boldsymbol{\varrho}_{m,k,i}, \mathbf{c}_{m,i}, \boldsymbol{\eta}_{m,i}, b_{0,0,i}, b_{0,m,i}, \mathbf{b}_{1,0,i}, \mathbf{b}_{1,m,i}, \boldsymbol{\gamma}_{0,i}, \boldsymbol{\gamma}_{m,i})\}_{m,k\in\{1,\ldots,M\}}$$

to be the collection of subject-specific parameters for $i = 1,\ldots,n$. For each $t = 1,\ldots,T$, $m = 1,\ldots,M$ and $i = 1,\ldots,n$ define the forward-variables $\alpha_i^t(m;\boldsymbol{\theta}_i) = P_{\theta_i}\left(Y_i^1,\ldots,Y_i^t, S_i^t = m\right)$ where P_{θ_i} denotes the distribution indexed by θ_i. We assume there is a finite latent state duration D, and any state durations exceeding D are artificially censored. Then it can be shown that the forward-variables satisfy the following recursions

$$\begin{aligned}
\alpha_i^1(m;\boldsymbol{\theta}_i) =& \delta_{m,i} r_{m,i}(1) P(Y_i^1|S_i^1 = m),\\
\alpha_i^2(m;\boldsymbol{\theta}_i) =& \delta_{m,i} r_{m,i}(2) \prod_{\tau=1}^{2} P(Y_i^\tau|S_i^\tau = m) + \sum_{\ell\neq m} \alpha_i^1(\ell;\boldsymbol{\theta}_i) Q_i(\ell,m) r_{m,i}(1) P(Y_i^2|S_i^2 = m),\\
\alpha_i^3(m;\boldsymbol{\theta}_i) =& \delta_{m,i} r_{m,i}(3) \prod_{\tau=1}^{3} P(Y_i^\tau|S_i^\tau = m) + \sum_{d=1}^{2}\sum_{\ell\neq m} \alpha_i^{3-d}(\ell;\boldsymbol{\theta}_i) Q_i(\ell,m) r_{m,i}(d) \prod_{\tau=4-d}^{3} P(Y_i^\tau|S_i^\tau = m),\\
&\vdots\\
\alpha_i^D(m;\boldsymbol{\theta}_i) =& \delta_{m,i} r_{m,i}(D) \prod_{\tau=1}^{D} P(Y_i^\tau|S_i^\tau = m) + \sum_{d=1}^{D-1}\sum_{\ell\neq m} \alpha_i^{D-d}(\ell;\boldsymbol{\theta}_i) Q_i(\ell,m) r_{m,i}(d) \prod_{\tau=D+1-d}^{D} P(Y_i^\tau|S_i^\tau = m),
\end{aligned}$$

where the terms on the right-hand-side of the above equations are dictated by $\boldsymbol{\theta}_i$ through the models posited previously; though we have suppressed this dependence in the notation. For $t = D+1,\ldots,T$ the forward-variables satisfy

$$\alpha_i^t(m;\boldsymbol{\theta}_i) = \sum_{\ell=1}^{M}\sum_{d=1}^{D}\left[\alpha_i^{t-d}(\ell;\boldsymbol{\theta}_i) Q_i(\ell,m) r_{m,i}(d) \prod_{\tau=t-d+1}^{t} P(Y_i^\tau|S_i^\tau = m)\right].$$

Thus, the likelihood for $\boldsymbol{\theta}_i$ is $\mathcal{L}_i(\boldsymbol{\theta}_i) = \sum_{m=1}^{M} \alpha_i^T(m;\boldsymbol{\theta}_i)$ and the likelihood for $\boldsymbol{\theta}_1,\ldots,\boldsymbol{\theta}_n$ is $\prod_{i=1}^{n}\mathcal{L}_i(\boldsymbol{\theta}_i)$. We construct estimators $\widehat{\boldsymbol{\theta}}_{i,n}$ of $\boldsymbol{\theta}_i$ by maximizing the log-likelihood.

For $m = 0,1,\dots,M$ let $\widehat{\mathbf{b}}_{1,m,i,T}$ denote the maximum likelihood estimator of $\mathbf{b}_{1,m,i}$. Subsequently, define $\widehat{\boldsymbol{\Omega}}_{m,0,n}, \widehat{\boldsymbol{\Omega}}_{m,1,n} = \arg\min_{\boldsymbol{\Omega}_{m,0}\in\mathbb{R}^q, \boldsymbol{\Omega}_{m,1}\in\mathbb{R}^{q\times d}} \sum_{i=1}^n \|\widehat{\boldsymbol{b}}_{1,m,i,T} - \boldsymbol{\Omega}_{m,0} - \boldsymbol{\Omega}_{m,1}\mathbf{W}_i\|^2$ to be the two-stage estimator of $\boldsymbol{\Omega}_{m,0}, \boldsymbol{\Omega}_{m,1}$. The estimators $\widehat{\boldsymbol{\Gamma}}_{m,0,n}, \widehat{\boldsymbol{\Gamma}}_{m,1,n}$, $\widehat{\boldsymbol{\Lambda}}_{m,\ell,0,n}$ and $\widehat{\boldsymbol{\Lambda}}_{m,\ell,1,n}$ of $\boldsymbol{\Gamma}_{m,0}, \widehat{\boldsymbol{\Gamma}}_{m,1}$, $\boldsymbol{\Lambda}_{m,\ell,0}$ and $\boldsymbol{\Lambda}_{m,\ell,1}$ are defined analogously.

Theoretical properties

Identifiability of the zero-inflated hidden Markov model

Proof. According to Proposition 3.1 in Gassiat et al (2013), if the state-dependent probability measures are linearly independent and the weight matrix has full rank, then the parameters in the hidden Markov model are identifiable up to label switching of the hidden states as soon as the transition matrix has full rank. In our case, define the M state-dependent probability measures $\mu_1,...,\mu_M$ by

$$\mu_j = \sum_{l=1}^{k} \psi_{jl}\phi_l \text{ for } j = 1,...,M,$$

where ϕ_1 is a point mass at zero, ϕ_l (l=2,...,M+1) are the M different Poisson distributions, and $\psi = [\psi_{jl}]$ is the weight matrix. Denote the structural zero proportion for the zero-inflated Poisson distribution in state 1 by p. Then the $M \times (M+1)$ weight matrix ψ is

$$\psi = \begin{bmatrix} p & 1-p & 0 & 0 & \dots & 0 \\ 0 & 0 & 1 & 0 & \dots & 0 \\ \vdots & & & \ddots & & \vdots \\ 0 & \dots & \dots & \dots & \dots & 1 \end{bmatrix},$$

where each row represents a latent state; column 1 represents the probability measure of the structural zeros; column 2 - column M+1 represent the M distinct Poisson distributions. It can be seen that ψ has rank M.

Proof of Lemma

Define the conditional log likelihood function for the subject-specific hidden semi-Markov model given initial state S_i^0 as

$$l_i^T(\theta_i, S_i^0) \triangleq \log P(Y_i^1,...,Y_i^T|S_i^0,\theta_i) = \sum_{t=1}^{T} \log P(Y_i^t|Y_i^0,...,Y_i^{t-1},S_i^0,\theta_i)$$

for subject $i = 1,...,n$. Under (A1), this is equivalent to the conditional log likelihood for a correspondingly reparameterized hidden Markov model with an expanded state space.

Under (A0) - (A4), the uniform convergence of the normalized log likelihood is proved in Proposition 2 in Douc et al (2004), i.e.,

$$\sup_{\theta_i} \sup_{S_i^0} |T^{-1} l_i^T(\theta_i, S_i^0) - l_i(\theta_i)| \to 0 \; a.s. \text{ as } T \to \infty \text{ for } i = 1,...,n,$$

where $l_i(\theta_i)$ is a deterministic asymptotic function such that

$$l_i(\theta_i) \triangleq \lim_{k\to\infty} E_{\theta_i^\star}\left[\log P(Y_i^t|Y_i^{-k},...,Y_i^{t-1},S_i^{-m},\theta_i)\right].$$

To show that $l_i(\theta_i)$ is maximized at $\theta_i = \theta_i^\star$ for $i = 1,...,n$,

$$\begin{aligned} & l_i(\theta_i) - l_i(\theta_i^\star) \\ =& \lim_{k\to\infty} E_{\theta_i^\star}\left[\log \frac{P(Y_i^t|Y_i^{-k},...,Y_i^{t-1},S_i^{-m},\theta_i)}{P(Y_i^t|Y_i^{-k},...,Y_i^{t-1},S_i^{-m},\theta_i^\star)}\right] \\ =& \lim_{k\to\infty} E_{\theta_i^\star}\left\{E_{\theta_i^\star}\left[\log \frac{P(Y_i^t|Y_i^{-k},...,Y_i^{t-1},S_i^{-m},\theta_i)}{P(Y_i^t|Y_i^{-k},...,Y_i^{t-1},S_i^{-m},\theta_i^\star)} | Y_i^{-k},...,Y_i^{t-1},S_i^{-m},\theta_i^\star\right]\right\}, \end{aligned}$$

where the inner conditional expectation is the Kullback-Leibler divergence and is maximized at $\theta_i = \theta_i^\star$.

By the identifiability assumption (A5), $\theta_i^\star$ is the unique maximizer of $l_i(\theta_i)$. Therefore, it follows that

$$\arg\max_{\theta_i} T^{-1} l_i^T(\theta_i, S_i^0) \to \arg\max_{\theta_i} l_i(\theta_i) \text{ with probability 1 as } T \to \infty.$$

Because $\arg\max_{\theta_i} T^{-1} l_i^T(\theta_i, S_i^0)$ is the MLE $\hat{\theta}_{i,T}$, we have

$$\hat{\theta}_{i,T} \to \theta_i^\star \text{ with probability 1 for all } i = 1, ..., n.$$

Proof of Theorem

It suffices to proof the theorem for $\widehat{\boldsymbol{\Omega}}_{m,n}$, which can then be naturally extended to $\widehat{\boldsymbol{\Gamma}}_{m,n}$ and $\widehat{\boldsymbol{\Lambda}}_{m,\ell,n}$ using the exact same approach, where $m, \ell = 1, ..., M$. By Assumptions (A0) - (A5), we have Lemma 1, which implies that the MLEs for subject-specific treatment effects $\widehat{\boldsymbol{b}}_{1,m,T}$ from the first-stage fitting are strongly consistent; this implies that

$$\text{Var}[\mathbf{vec}(\widehat{\boldsymbol{b}}_{1,m,T})_i] \longrightarrow 0 \quad \text{for all } i = 1, ..., qn,\ m = 1, ..., M \text{ as } T \longrightarrow \infty.$$

Recall that the linear regression model we fit in the second stage is of the form

$$\mathbf{vec}(\widehat{\boldsymbol{b}}_{1,m,T})_i = \tilde{\boldsymbol{B}}_i' \mathbf{vec}(\boldsymbol{\Omega}_m) + v_{m,i} \text{ for } m = 1, ..., M,\ i = 1, ..., qn$$

where $\tilde{\boldsymbol{B}} \triangleq \boldsymbol{I} \otimes \tilde{\boldsymbol{W}} \in \mathbb{R}^{qn \times q(d+1)}$ the ith row of which is $\tilde{\boldsymbol{B}}_i$; $v_{m,i} = u_{m,i} + e_{m,i}$; $e_{m,i}$ and is independent across all indices with mean 0 and variance σ^2; $u_{m,i}$ and furthermore is independent across all indices with mean 0 and variance $\text{Var}[\mathbf{vec}(\widehat{b}_{1,m,T})_i]$. In the corresponding matrix notation,

$$\mathbf{vec}(\widehat{\boldsymbol{b}}_{1,m,T}) = \tilde{\boldsymbol{B}}\, \mathbf{vec}(\boldsymbol{\Omega}_m) + \boldsymbol{v}_m \text{ for } m = 1, ..., M.$$

Consider the true linear model for $\boldsymbol{b}_{1,m}$ as

$$\mathbf{vec}(\boldsymbol{b}_{1,m}) = \tilde{\boldsymbol{B}}\, \mathbf{vec}(\boldsymbol{\Omega}_m^*) + \boldsymbol{e}_m \text{ for } m = 1, ..., M,$$

where $\mathbf{vec}(\widehat{\boldsymbol{b}}_{1,m,T}) = \mathbf{vec}(\boldsymbol{b}_{1,m}) + \boldsymbol{u}_m$ such that $\boldsymbol{u}_{m,k}$ mimics the measurement error process depending only on the first-stage fit. We want to show $\mathbf{vec}(\widehat{\boldsymbol{\Omega}}_{m,n}) \xrightarrow{p} \mathbf{vec}(\boldsymbol{\Omega}_m^*)$ as $n \longrightarrow \infty$. Because the first-stage subject covariates and environmental factors are not correlated with the second-stage baseline characteristics, we have $\boldsymbol{u}_m \perp\!\!\!\perp \boldsymbol{e}_m$, $\boldsymbol{u}_m \perp\!\!\!\perp \tilde{\boldsymbol{B}}$, and $\boldsymbol{e}_m \perp\!\!\!\perp \tilde{\boldsymbol{B}}$, so that $E(\tilde{\boldsymbol{B}}^T \boldsymbol{u}_m) = 0$, $E(\tilde{\boldsymbol{B}}^T \boldsymbol{e}_m) = 0$ for $m = 1, ..., M$.

We also assume that $\lim_{n\to\infty} \frac{1}{n} \tilde{\boldsymbol{W}}^T \tilde{\boldsymbol{W}} = \mathbf{H}$ where $\mathbf{H}$ is a positive definite matrix. Since $E\left(\frac{\tilde{\boldsymbol{B}}^T \boldsymbol{v}_m}{n}\right) = E\left(\frac{\tilde{\boldsymbol{B}}^T (\boldsymbol{u}_m + \boldsymbol{e}_m)}{n}\right) = 0$ and $E(v_{m,i}^2) = E(u_{m,i}^2) + E(e_{m,i}^2) = \text{Var}[\mathbf{vec}(\widehat{b}_{1,m,T})_i] + \sigma^2 \xrightarrow{p} \sigma^2$, then $Var(v_{m,i}) \le g\sigma^2$ for all $i = 1, ..., nq$ when T is large, where $g > 1$ is some fixed positive constant. Then

$$Var\left(\frac{\tilde{\boldsymbol{B}}^T \boldsymbol{v}_m}{n}\right) = \frac{\tilde{\boldsymbol{B}}^T Var(\boldsymbol{v}_m) \tilde{\boldsymbol{B}}}{n^2} \le \frac{g\sigma^2}{n} \frac{\tilde{\boldsymbol{B}}^T \tilde{\boldsymbol{B}}}{n} = \frac{g\sigma^2}{n} \left[\mathbf{I} \otimes \frac{1}{n} \tilde{\boldsymbol{W}}^T \tilde{\boldsymbol{W}}\right] \to 0 \quad \text{as } n \to \infty,$$

which implies that $\frac{\tilde{\boldsymbol{B}}^T \boldsymbol{v}_m}{n} \xrightarrow{p} 0$. Therefore, we can show

$$\begin{aligned}
\mathbf{vec}(\widehat{\boldsymbol{\Omega}}_{m,n}) &= \left(\frac{\tilde{\boldsymbol{B}}^T \tilde{\boldsymbol{B}}}{n}\right)^{-1} \frac{\tilde{\boldsymbol{B}}^T \mathbf{vec}(\widehat{\boldsymbol{b}}_{1,m,T})}{n} \\
&= \left(\frac{\tilde{\boldsymbol{B}}^T \tilde{\boldsymbol{B}}}{n}\right)^{-1} \frac{\tilde{\boldsymbol{B}}^T (\tilde{\boldsymbol{B}} \mathbf{vec}(\boldsymbol{\Omega}_m^*) + \boldsymbol{v}_m)}{n} \\
&= \mathbf{vec}(\boldsymbol{\Omega}_m^*) + \left(\frac{\tilde{\boldsymbol{B}}^T \tilde{\boldsymbol{B}}}{n}\right)^{-1} \frac{\tilde{\boldsymbol{B}}^T \boldsymbol{v}_m}{n} \\
&\xrightarrow{p} \mathbf{vec}(\boldsymbol{\Omega}_m^*)
\end{aligned}$$

for $m = 1, ..., M$ as $n \longrightarrow \infty$. Thus, $\mathbf{vec}(\widehat{\boldsymbol{\Omega}}_{m,n}) \xrightarrow{p} \mathbf{vec}(\boldsymbol{\Omega}_m^*)$ for $m = 1, ..., M$ as $n \longrightarrow \infty$.

By Lindeberg-Feller Central Limit Theorem, $\sqrt{n}(\mathbf{vec}(\widehat{\boldsymbol{\Omega}}_{m,n}) - \mathbf{vec}(\boldsymbol{\Omega}_m^*))$ converges in distribution to a multivariate Gaussian distribution with mean $\mathbf{0}$ and covariance

$$(\mathbf{I} \otimes \mathbf{H})^{-1} \left[\frac{1}{n} \tilde{\boldsymbol{B}}^T \mathbf{diag}\{E(v_{m,i}^2)\} \tilde{\boldsymbol{B}}\right] (\mathbf{I} \otimes \mathbf{H})^{-1},$$

where $\mathbf{diag}\{E(v_{m,i}^2)\}$ is a diagonal matrix with elements $\{E(v_{m,i}^2)\}$.

By the consistency of the sandwich variance estimator,

$$\left(\left[\frac{\tilde{\boldsymbol{B}}^T\tilde{\boldsymbol{B}}}{n}\right]^{-1}\left[\frac{1}{n}\tilde{\boldsymbol{B}}^T\mathbf{diag}\left\{[\mathbf{vec}(\widehat{\boldsymbol{b}}_{1,m,T})_i-\tilde{\boldsymbol{B}}_i^T\mathbf{vec}(\boldsymbol{\Omega}_m^*)]^2\right\}\tilde{\boldsymbol{B}}\right]\left[\frac{\tilde{\boldsymbol{B}}^T\tilde{\boldsymbol{B}}}{n}\right]^{-1}\right)$$
$$\to(\mathbf{I}\otimes\mathbf{H})^{-1}\left[\frac{1}{n}\tilde{\boldsymbol{B}}^T\mathbf{diag}\{E(v_{m,i}^2)\}\tilde{\boldsymbol{B}}\right](\mathbf{I}\otimes\mathbf{H})^{-1}$$

Thus, by Slutsky's Theorem, the following quantity converges in distribution to a multivariate Gaussian distribution with mean zero and identity covariance,

$$\left(\left[\frac{\tilde{\boldsymbol{B}}^T\tilde{\boldsymbol{B}}}{n}\right]^{-1}\left[\frac{1}{n}\tilde{\boldsymbol{B}}^T\mathbf{diag}\left\{[\mathbf{vec}(\widehat{\boldsymbol{b}}_{1,m,T})_i-\tilde{\boldsymbol{B}}_i^T\mathbf{vec}(\widehat{\boldsymbol{\Omega}}_{m,n})]^2\right\}\tilde{\boldsymbol{B}}\right]\left[\frac{\tilde{\boldsymbol{B}}^T\tilde{\boldsymbol{B}}}{n}\right]^{-1}\right)^{-1/2}\times\sqrt{n}\left\{\mathbf{vec}(\widehat{\boldsymbol{\Omega}}_{m,n})-\mathbf{vec}(\boldsymbol{\Omega}_m^*)\right\}.$$

Simulation experiments

We study the finite sample performance of the proposed two-stage estimator using a suite of simulation experiments. Across these experiments, we consider three different pathways for the treatment effect: (Scenario I) a change in the state-dependent means and the proportion of zeros; (Scenario II) a change in the latent state durations; and (Scenario III) a change in the state-transition probabilities. In each scenario, we simulate minute-by-minute activity counts during a two week period for $n=10$ and $n=20$ subjects. Each simulated trajectory is divided into an initial one-week baseline phase followed by a one-week treatment period; thus, each patient has 10,080 observations per period. The activity counts are generated using a three state zero-inflated Poisson hidden semi-Markov model, with the maximum latent state duration set to be 20. The initial state probabilities are distributed as follows: let $U_1,U_2\sim_{i.i.d.}$ Uniform$(0,1)$ and set $\delta_1=\min(U_1,U_2)$, $\delta_2=\max(U_1,U_2)-\delta_1$, and $\delta_3=1-\delta_1-\delta_2$. We generate subject covariates so that half of the subjects are male, and set the baseline Poisson means for the male subjects to be 5% less than the female subjects while the baseline zero proportion is 5% larger than the female subjects. Furthermore, for all subjects (regardless of sex) the Poisson means are 5% less at night than during the day and the zero proportions are 5% larger at night than during the day. These proportions are consistent with the accelerometer data collected in the study of the treatment effect of meloxicam.

In Scenario I, treatment increases the log mean activity in all the latent states by 10% and decreases the log odds of zero in state 1 by 10%, so that

$$\begin{aligned}\log\left\{\frac{p_i^t(\mathbf{z}^t,\mathbf{x}^t)}{1-p_i^t(\mathbf{z}^t,\mathbf{x}^t)}\right\} &= b_{0,0,i}+0.05\times\mathbf{I}\{\text{Male}\}_i-0.1\times\mathbf{I}\{\text{Trt}\}_i^t+0.05\times\mathbf{I}\{\text{Night}\}_i^t,\\ \log\left\{\lambda_i^t(m,\mathbf{z}^t,\mathbf{x}^t)\right\} &= b_{0,m,i}-0.05\times\mathbf{I}\{\text{Male}\}_i+0.1\times\mathbf{I}\{\text{Trt}\}_i^t-0.05\times\mathbf{I}\{\text{Night}\}_i^t,\end{aligned}$$

for $m=1,2,3$, where $b_{0,0,i}\overset{iid}{\sim}N(0,0.1^2)$, $b_{0,1,i}\overset{iid}{\sim}N\left\{\log(50),0.1^2\right\}$, $b_{0,2,i}\overset{iid}{\sim}N\left\{\log(300),0.1^2\right\}$, and $b_{0,3,i}\overset{iid}{\sim}N\left\{\log(700),0.1^2\right\}$. Let $N_{[a,b]}(\mu,\sigma^2)$ denote a normal distribution with mean μ and variance σ^2 truncated to $[a,b]$. In this scenario, we assume that both the transition probabilities and the latent state durations are unaffected by the treatment and that $\mathbf{Q}_i(1,2)\overset{iid}{\sim}N_{[0,1]}(0.5,0.01)$, $\mathbf{Q}_i(2,1)\overset{iid}{\sim}N_{[0,1]}(0.7,0.01)$, $\mathbf{Q}_i(3,1)\overset{iid}{\sim}N_{[0,1]}(0.7,0.01)$, so that the transition matrix for subject i is

$$\begin{bmatrix}0 & \mathbf{Q}_i(1,2) & 1-\mathbf{Q}_i(1,2)\\ \mathbf{Q}_i(2,1) & 0 & 1-\mathbf{Q}_i(2,1)\\ \mathbf{Q}_i(3,1) & 1-\mathbf{Q}_i(3,1) & 0\end{bmatrix}.$$

This setup mimics the real accelerometer data where state 1 (lowest activity level) dominates the other latent states. We do not allow transition back to the same state as the latent state durations modeled separatetly; in Scenario 1, the latent state durations are uniformly distributed on $\{1,2,\ldots,20\}$ for each state.

In Scenario II, we assume that the treatment increases the log mean activity in state 3 by 10% but decreases the log mean

activity in state 1 and 2 by 10%. Further, the treatment increases the log odds of zero in state 1 by 10%, such that

$$\begin{aligned}
\log\left\{\frac{p_i^t(\mathbf{z}^t,\mathbf{x}^t)}{1-p_i^t(\mathbf{z}^t,\mathbf{x}^t)}\right\} &= b_{0,0,i}+0.05\times\mathbf{I}\{\text{Male}\}_i+0.1\times\mathbf{I}\{\text{Trt}\}_i^t+0.05\times\mathbf{I}\{\text{Night}\}_i^t,\\
\log\left\{\lambda_i^t(1,\mathbf{z}^t,\mathbf{x}^t)\right\} &= b_{0,1,i}-0.05\times\mathbf{I}\{\text{Male}\}_i-0.1\times\mathbf{I}\{\text{Trt}\}_i^t-0.05\times\mathbf{I}\{\text{Night}\}_i^t,\\
\log\left\{\lambda_i^t(2,\mathbf{z}^t,\mathbf{x}^t)\right\} &= b_{0,2,i}-0.05\times\mathbf{I}\{\text{Male}\}_i-0.1\times\mathbf{I}\{\text{Trt}\}_i^t-0.05\times\mathbf{I}\{\text{Night}\}_i^t,\\
\log\left\{\lambda_i^t(3,\mathbf{z}^t,\mathbf{x}^t)\right\} &= b_{0,3,i}-0.05\times\mathbf{I}\{\text{Male}\}_i+0.1\times\mathbf{I}\{\text{Trt}\}_i^t-0.05\times\mathbf{I}\{\text{Night}\}_i^t,
\end{aligned}$$

where $b_{0,0,i} \overset{iid}{\sim} N(0,0.01)$, $b_{0,1,i} \overset{iid}{\sim} N(\log(50),0.01)$, $b_{0,2,i} \overset{iid}{\sim} N(\log(300),0.01)$, $b_{0,3,i} \overset{iid}{\sim} N(\log(700),0.01)$. In this scenario, treatment will increase activity in the highest level state but make the subject less agitated in lower level activity states. In this scenario, both the transition probabilities and the latent state durations are unaffected by the treatment and match the settings of Scenario I.

In Scenario III, we assume that treatment accelerates the duration time by 10% in each latent state, so that subjects are more likely to actively switch between states when they are on treatment, i.e.,

$$r_{m,i}\left(v;\mathbf{x}^t,\mathbf{z}^t\right)=\exp\left(0.1\times\mathbf{I}\{\text{Trt}\}_i^t\right)\int_v^{v+1} f_{m,i}\left\{\exp\left(0.1\times\mathbf{I}\{\text{Trt}\}_i^t\right)u\right\}du,$$

for $m=1,2,3$, where $f_{1,i}$, $f_{2,i}$, $f_{3,i}$ are the exponential densities with rate parameters $\xi_{1,i}$, $\xi_{2,i}$, $\xi_{3,i}$, such that $\xi_{1,i} \overset{iid}{\sim} N(5,0.1^2)$, $\xi_{2,i} \overset{iid}{\sim} N(3,0.1^2)$, and $\xi_{3,i} \overset{iid}{\sim} N(2,0.1^2)$. This setup also mimics the study of the treatment effect of the meloxicam in that the expected duration in state 1 is longer than in other states. We assume that the treatment does not affect the state-dependent Poisson means and zero proportion so that

$$\begin{aligned}
\log\left\{\frac{p_i^t(\mathbf{z}^t,\mathbf{x}^t)}{1-p_i^t(\mathbf{z}^t,\mathbf{x}^t)}\right\} &= b_{0,0,i}+0.05\times\mathbf{I}\{\text{Male}\}_i+0.05\times\mathbf{I}\{\text{Night}\}_i^t\\
\log\left\{\lambda_i^t(m,\mathbf{z}^t,\mathbf{x}^t)\right\} &= b_{0,m,i}-0.05\times\mathbf{I}\{\text{Male}\}_i-0.05\times\mathbf{I}\{\text{Night}\}_i^t,
\end{aligned}$$

for all $m=1,2,3$, where $b_{0,0,i} \overset{iid}{\sim} N(0,0.1^2)$, $b_{0,1,i} \overset{iid}{\sim} N\left\{\log(50),0.1^2\right\}$, $b_{0,2,i} \overset{iid}{\sim} N\left\{\log(300),0.1^2\right\}$, $b_{0,3,i} \overset{iid}{\sim} N\{\log(700),0.01\}$. The transition probabilities are the same as in Scenario I.

In Scenario IV, we assume that treatment affects the state transition probabilities by increasing the odds of moving to the next most active state by 10% so that

$$\begin{aligned}
\log\left\{\frac{Q_i(1,3;\mathbf{x}^t,\mathbf{z}^t)}{Q_i(1,2;\mathbf{x}^t,\mathbf{z}^t)}\right\} &= \eta_{1,i}+0.1\times\mathbf{I}\{\text{Trt}\}_i^t,\\
\log\left\{\frac{Q_i(2,3;\mathbf{x}^t,\mathbf{z}^t)}{Q_i(2,1;\mathbf{x}^t,\mathbf{z}^t)}\right\} &= \eta_{2,i}+0.1\times\mathbf{I}\{\text{Trt}\}_i^t,\\
\log\left\{\frac{Q_i(3,2;\mathbf{x}^t,\mathbf{z}^t)}{Q_i(3,1;\mathbf{x}^t,\mathbf{z}^t)}\right\} &= \eta_{3,i}+0.1\times\mathbf{I}\{\text{Trt}\}_i^t,
\end{aligned}$$

where $\eta_{1,i} \overset{iid}{\sim} N(0,0.1^2)$, $\eta_{2,i} \overset{iid}{\sim} N(-0.5,0.1^2)$, $\eta_{1,i} \overset{iid}{\sim} N(-0.5,0.1^2)$. The latent state duration are $r_{m,i}(v)=\int_v^{v+1} f_{m,i}\{u\}du$, for $m=1,2,3$, where $f_{1,i}$, $f_{2,i}$, $f_{3,i}$ are the exponential densities with rate parameters $\xi_{1,i}$, $\xi_{2,i}$, $\xi_{3,i}$ respectively, such that $\xi_{1,i} \overset{iid}{\sim} N(5,0.1^2)$, $\xi_{2,i} \overset{iid}{\sim} N(3,0.1^2)$, $\xi_{3,i} \overset{iid}{\sim} N(2,0.1^2)$. The state-dependent Poisson means and zero proportion are as in Scenario III.

We compare the proposed estimator with the baseline treatment effect estimator constructed using a generalized estimating equation (GEE). This is equivalent to the repeated-measures ANOVA model as the data are complete and balanced. In the GEE model, denote the new response variable in the model by $\tilde{Y}_i^\tau$, which is the average activity count for subject i on day $\tau=1,\ldots,14$. Define $\tilde{\mathbf{Y}}_i \triangleq [\tilde{Y}_i^1,...,\tilde{Y}_i^{14}]$. Let $E(\tilde{\mathbf{Y}}_i|\tilde{\mathbf{X}}_i)=\tilde{\mathbf{X}}_i\boldsymbol{\beta}$ and $\text{var}(\tilde{\mathbf{Y}}_i|\tilde{\mathbf{X}}_i)=\mathbf{V}_i$, where $\tilde{\mathbf{X}}_i\in\mathbb{R}^{14\times 3}$ whose τ^{th} row is $[1,\mathbf{I}\{\text{Trt}\}_i^\tau,\mathbf{I}\{\text{Male}\}_i]$; $\boldsymbol{\beta}=[\beta_0,\beta_1,\beta_2]$ so that β_1 represents the treatment effect; the working covariance $\mathbf{V}_i$ is assumed to be compound symmetric.

Table 1 shows the mean and standard error for the estimated treatment effect estimators in the proposed method using 500 Monte Carlo replications. The estimators are approximately unbiased in all four scenarios and the standard error is small even with a moderate number of samples owing to within-subject pooling across observations. Table 2 shows the mean and standard error for the estimated treatment effect in the baseline GEE model. The GEE method manages to detect the treatment effect in Scenarios I and IV, where there is an increase in the marginal mean activity counts under treatment; however, it fails to detect the nonzero conditional treatment effect in different latent states using an $\alpha=0.05$ level test in Scenario II and III because there is only a small difference in the marginal mean activity counts.

Table 1. Mean (standard error) of the treatment effect estimators from the proposed hidden semi-markov model in Scenarios I, II, III, and IV for each latent state based on 500 Monte Carlo simulations.

Parameter	True effect	n=10<br>Estimated treatment effect (s.e.)	n=20<br>Estimated treatment effect (s.e.)
**Scenario I**			
State 1: $\widehat{\Omega}_{0,0}$	-0.1	-.0997 (.0159)	-.0994 (.0113)
State 1: $\widehat{\Omega}_{1,0}$	0.1	.0999 (.0015)	.0999 (.0011)
State 2: $\widehat{\Omega}_{2,0}$	0.1	.1000 (.0007)	.1000 (.0005)
State 3: $\widehat{\Omega}_{3,0}$	0.1	.1000 (.0007)	.1000 (.0005)
**Scenario II**			
State 1: $\widehat{\Omega}_{0,0}$	0.1	.0994 (.0159)	.1002 (.0116)
State 1: $\widehat{\Omega}_{1,0}$	-0.1	-.1000 (.0017)	-.1001 (.0012)
State 2: $\widehat{\Omega}_{2,0}$	-0.1	-.1001 (.0008)	-.1000 (.0006)
State 3: $\widehat{\Omega}_{3,0}$	0.1	.0999 (.0007)	.1000 (.0005)
**Scenario III**			
State 1: $\widehat{\Gamma}_{1,0}$	0.1	.0931 (.0177)	.0931 (.0131)
State 2: $\widehat{\Gamma}_{2,0}$	0.1	.0931 (.0216)	.0920 (.0158)
State 3: $\widehat{\Gamma}_{3,0}$	0.1	.0935 (.0222)	.0952 (.0167)
**Scenario IV**			
State 1: $\widehat{\Lambda}_{1,3,0}$	0.1	.1006 (.0414)	.1022 (.0290)
State 2: $\widehat{\Lambda}_{2,3,0}$	0.1	.0983 (.0486)	.1006 (.0346)
State 3: $\widehat{\Lambda}_{3,2,0}$	0.1	.0995 (.0447)	.1001 (.0321)

Table 2. Mean (standard error) of the treatment effect estimators from the baseline GEE model for Scenario I, II, III, and IV via 500 Monte Carlo simulations.

Scenario	n=10<br>Estimated treatment effect (s.e.)	n=20<br>Estimated treatment effect (s.e.)
I	19.77 (1.86)	19.57 (1.26)
II	0.46 (1.94)	0.54 (1.24)
III	1.21 (1.91)	1.16 (1.33)
IV	6.02 (2.08)	5.88 (1.48)
